# Thenar oxygen saturation (StO_2_) alterations during a spontaneous breathing trial predict extubation failure

**DOI:** 10.1186/s13613-020-00670-y

**Published:** 2020-05-11

**Authors:** Jaume Mesquida, Guillem Gruartmoner, Cristina Espinal, Jordi Masip, Caroline Sabatier, Ana Villagrá, Hernando Gómez, Michael Pinsky, Francisco Baigorri, Antonio Artigas

**Affiliations:** 1 Critical Care Department, Parc Taulí Hospital Universitari. Institut d’Investigació i Innovació Parc Taulí (I3PT), Parc Taulí, 1, 08208 Sabadell, Spain; 2 Departament de Medicina, Universitat Autònoma de Barcelona, Bellaterra, Spain; 3 Réanimation Polyvalente, Centre Hospitalier de Pau, Pau, France; 4 Critical Care Department, Osakidetza Basque Health Service, Alava University Hospital, Vitoria-Gasteiz, Spain; 5 Department of Critical Care Medicine, University of Pittsburgh Medical Center, Pittsburgh, PA USA; 6 Center for Critical Care Nephrology, University of Pittsburgh, Pittsburgh, PA USA; 7 CIBER de Enfermedades Respiratorias, Sabadell, Spain

**Keywords:** Near-infrared spectroscopy, Tissue oxygenation, Microcirculation, Regional blood flow, Mechanical ventilation, Weaning

## Abstract

**Background:**

Weaning from mechanical ventilation (MV) is a cardiovascular stress test. Monitoring the regional oxygenation status has shown promising results in predicting the tolerance to spontaneously breathe in the process of weaning from MV. Our aim was to determine whether changes in skeletal muscle oxygen saturation (StO_2_) measured by near-infrared spectroscopy (NIRS) on the thenar eminence during a vascular occlusion test (VOT) can be used to predict extubation failure from mechanical ventilation.

**Methods:**

We prospectively studied 206 adult patients with acute respiratory failure receiving MV for at least 48 h from a 30-bed mixed ICU, who were deemed ready to wean by their physicians. Patients underwent a 30-min spontaneous breathing trial (SBT), and were extubated according to the local protocol. Continuous StO_2_ was measured non-invasively on the thenar eminence. A VOT was performed prior to and at 30 min of the SBT (SBT_30_). The rate of StO_2_ deoxygenation (DeO_2_), StO_2_ reoxygenation (ReO_2_) rate and StO_2_ hyperemic response to ischemia (*H*_AUC_) were calculated.

**Results:**

Thirty-six of the 206 patients (17%) failed their SBT. The remainder 170 patients (83%) were extubated. Twenty-three of these patients (13.5%) needed reinstitution of MV within 24 h. Reintubated patients displayed a lower *H*_AUC_ at baseline, and higher relative changes in their StO_2_ deoxygenation rate between baseline and SBT_30_ (DeO_2_ Ratio). A logistic regression-derived StO_2_ score, combining baseline StO_2_, *H*_AUC_ and DeO_2_ ratio, showed an AUC of 0.84 (95% CI 0.74–0.91) for prediction of extubation failure.

**Conclusions:**

Extubation failure was associated to baseline and dynamic StO_2_ alterations during the SBT. Monitoring StO_2_-derived parameters might be useful in predicting extubation outcome.

## Background

Weaning from ventilatory support is a challenge for critical care clinicians. Even in those patients who succeed a spontaneous breathing trial (SBT), failure of planned extubation occurs in up to 20% [1–5]. Importantly, failed extubation is associated with increased hospital mortality, prolonged ICU and hospital stays, and increased need for tracheostomy [6, 7]. Despite many respiratory and hemodynamic variables for predicting weaning success have been evaluated, few parameters have substantial predictive power [7, 8], and new predictive tools are needed.

The SBT is a cardiovascular stress test, and failure to wean from mechanical ventilation (MV) often reflects cardiovascular insufficiency to cope with the increased oxygen cost of breathing [9, 10]. Since the augmented oxygen cost of breathing is met by increases in respiratory muscle blood flow, diversion of flow away from “non-vital” tissues might occur, potentially causing hypoperfusion in areas such as the splanchnic and the peripheral circulation [11–15]. In fact, alterations in splanchnic and peripheral circulation, assessed by different technologies, have been associated with failure to tolerate an SBT [12–16]. In a preliminary study, our group demonstrated that changes in skeletal muscle oxygenation (StO_2_) measured non-invasively on the thenar eminence by near-infrared spectroscopy (NIRS) were associated with the outcome of a 30-min SBT [16].

In the present study, we hypothesized that regional oxygenation alterations within the SBT would be associated with extubation failure after a clinically successful SBT.

## Methods

### Setting

This prospective observational study was conducted in a 30-bed medical-surgical intensive care unit at a university hospital. The local Ethics Committee (CEIC 2008/554) approved the study. Informed consent was obtained from the patient or their next of kin prior to the study initiation. This study is presented according to the STROBE recommendations for reporting observational studies [17].

### Patients and data collection

We included adult patients (≥ 18-year old) receiving invasive mechanical ventilatory support for > 48 h and considered ready to wean by their physicians, according to the local protocol, and who never experienced any previous attempt of separation from MV. Eligibility to perform a weaning trial included partial or complete recovery from the underlying cause of acute respiratory failure; adequate gas exchange, as indicated by a partial pressure of arterial oxygen (PaO_2_) > 60 mmHg to a fraction of inspired oxygen (FiO_2_) < 0.4, with a positive end-expiratory pressure (PEEP) < 5 cmH_2_O; adequate cough, with absence of excessive tracheobronchial secretion; core temperature < 38 °C; hemoglobin > 8 g/dL; stable cardiovascular status (heart rate < 120 beats min^−1^, systolic blood pressure 90–160 mmHg), with no need for vasoactive agents; adequate level of consciousness (awake, alert and aware of their surroundings), with no further need for sedative agents. Patients were also tested for adequate pulmonary function, defined as a rapid shallow breathing index < 100 breaths min^−1^ L^−1^, and a maximal inspiratory pressure (MIP) < − 20 cmH_2_O. Patients meeting these criteria were considered ready to wean, and were eligible for a subsequent spontaneous breathing trial.

Exclusion criteria included trauma in both upper limbs, and hematoma or skin lesions at the thenar eminence that could hinder placement of NIRS sensor probe. Patients with altered level of consciousness that could lead to central hypoventilation and/or impaired secretions’ management were excluded. Patients in whom their medical team decided to indicate preventive non-invasive ventilation following extubation were also excluded.

### Study protocol

After inclusion, patients underwent a spontaneous breathing trial (SBT) for 30 min, defined as assisted spontaneous breathing with continuous positive airway pressure (CPAP) of 5 cm H_2_O, or a T-tube trial, as prescribed by their medical team. Patients were in a semi-recumbent position, and FiO_2_ was kept constant during the trial.

The evaluation criteria for SBT failure was defined as the presence of one or more of the following criteria during the trial: respiratory rate (RR) > 35 breaths/min for 5 min or longer; arterial pulse oximetry saturation (SpO_2_) < 90%, heart rate (HR) > 140 beats/min or sustained increase or decrease in HR > 20%; systolic blood pressure > 180 mmHg or < 90 mmHg; increased anxiety and diaphoresis. Decision to remove the endotracheal tube was made independently of the study investigators by the attending physicians, who did not have access to the StO_2_ data. Weaning success was defined as patient remaining free of MV for > 24 h after passing the SBT. Extubation failure was defined as the need for reinstitution of MV, either invasive or non-invasive, within 24 h following extubation.

### Clinical parameters and outcomes

We collected demographic data: age, sex, diagnosis, and days on mechanical ventilation. Hemodynamic, respiratory and oxygenation variables were monitored continuously and recorded just before starting and at 30 min into the SBT. HR and mean arterial pressure (MAP) were recorded by routine bedside monitoring (Monitor Intellivue MP 70; Phillips Medizinsystems, Boeblingen, Germany). RR, tidal volume (Vt), minute ventilation (*V*_E_), FiO_2_ and SpO_2_ were recorded at start and 30 min into the SBT. Arterial and/or central venous blood gas analyses were made when an arterial line and/or a central venous line was in place, respectively (ABL 700 series; Radiometer Medical, Copenhagen, Denmark).

The primary outcome of interest was extubation failure.

### NIRS measurements

Tissue oxygen saturation (StO_2_) was recorded continuously using the InSpectra 650 Tissue Spectrometer (Hutchinson Tech., Hutchinson, Minnessotta). This technology uses a wide gap 40-nm second derivative spectroscopic method, with measurements at four different wavelengths (680, 720, 760 and 800 nm), and has been previously validated for estimating local hemoglobin oxygen saturation in tissues [18]. The StO_2_ 15-mm optical surface probe was placed on intact skin on the thenar eminence never placed adjacent to the site of radial artery cannulation. The InSpectra tissue spectrometer also measures relative hemoglobin concentration, presented as the tissue hemoglobin index (THI).

**Fig. 1:**
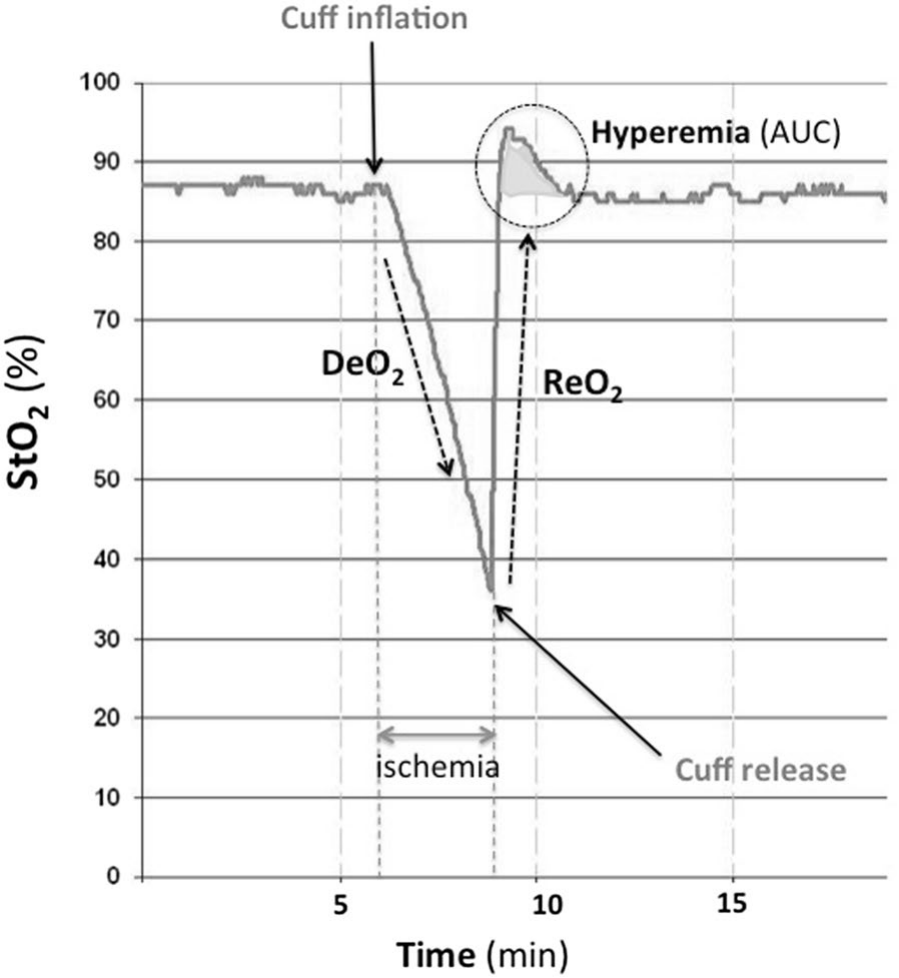
StO_2_ response to the Vascular Occlusion Test (VOT). The response to transient ischemia generates three main parameters in the continuous StO_2_ recording: the StO_2_ deoxygenation rate (DeO_2_), the StO_2_ reoxygenation rate (ReO_2_), and the hyperemic response to ischemia, which is computed as the area under the curve of the hyperemic response (*H*_AUC_) as referred to steady-state StO_2_

*Vascular Occlusion Test (VOT)* The VOT was performed as previously described [19, 20]. A blood pressure cuff (Portable Tourniquet System; Delfi Medical, Vancouver) was placed on the forearm, rapidly inflated 40 mmHg above systolic pressure, and kept inflated until StO_2_ decreased to 40%. Then, the cuff was rapidly deflated. The resulting deoxygenation (DeO_2_) and reoxygenation (ReO_2_) slopes are reported as change in saturation over time (Fig. 1). Hyperemic response following the reoxygenation is reported as an area under the curve (*H*_AUC_). We performed a VOT at the beginning of the SBT and again at 30 min into the SBT. NIRS-derived thenar muscle oxygen consumption (nirVO_2_) was calculated as described by Skarda [21]: nirVO_2_ = (DeO_2_ slope)^−1^/[(THI_start_ + THI_end_)/2]. Relative changes in StO_2_-derived parameters were calculated as the quotient between values at minute 30 and baseline (DeO_2_ ratio, nirVO_2_ ratio and ReO_2_ ratio). Absolute StO_2_- and VOT-derived variables were obtained using the InSpectra Research Software^®^ v4.01 (Hutchinson Technology). Two researchers (JM and JM) separately analyzed all the registers, and a database was constructed. When discrepancies were observed, the two researchers performed a third joint analysis.

### Sample size calculation

Accepting an alpha risk of 0.05 and a beta risk of 0.20 in a two-sided test, estimating an SBT failure rate of 20%, and an extubation failure rate of 15%, we calculated that 200 patients were necessary to detect a positive likelihood ratio (LR +) of the test equal to or greater than 5.

### Statistical analysis

Statistical analysis was performed by means of IBM SPSS statistics 20.0 software (IBM Corporation). Normal distribution of the studied variables was confirmed using the Kolmogorov–Smirnov test. Accordingly, continuous variables were expressed as mean ± standard deviation (SD), and categorical variables were expressed as absolute number and proportions (%). Chi square and Student’s *t* test were used to compare extubation success and failure groups. Student’s *t* test for paired measurements was used to analyze changes over time. Bivariate logistic regression analysis was used to obtain independent predictors of extubation success, and logistic regression models were used to create a predictive StO_2_-score. Statistical significance was defined as *p *< 0.05 (two-tailed test).

## Results

Two hundred-and-six patients were studied. Thirty-six patients (17.5%) failed the SBT, and were excluded of the final analysis. One hundred-and-seventy patients passed the SBT and were extubated. Twenty-three (13.5%) of these patients failed extubation and required re-instauration of MV within 24 h. The suspected etiology of extubation failure was mainly increased respiratory muscle load (52%), and cardiac failure (35%) (Additional file 1: Table S1). No baseline differences in demographic, hemodynamic, and respiratory variables were observed when comparing extubation success and extubation failure groups (Table 1). Hemodynamic and respiratory changes within the SBT were similar in both groups (Table 1).

**Table 1:** Characteristics of the patients who succeed the spontaneous breathing trial (SBT)

	Extubation success (*n* = 147)		Extubation failure (*n* = 23)	
Age (years)	66 ± 14		67 ± 12	
Days on MV (*n*)	7 ± 5		8 ± 6	
Pre-existent comorbidities (%)				
Diabetes mellitus	33		35	
Hypertension	57		48	
Cardiac disease	23		22	
COPD	16		30	
Etiology of acute respiratory failure (%)				
Septic shock	29		26	
Heart failure	17		26	
Respiratory	20		26	
Trauma	6		0	
Other	29		22	

	Baseline	Minute 30	Baseline	Minute 30
HR (beats/min)	88 ± 16	91 ± 16^†^	83 ± 17	87 ± 18^†^
MAP (mmHg)	82 ± 13	85 ± 15^†^	79 ± 14	80 ± 12
RR (resp/min)	19 ± 4	23 ± 5^†^	18 ± 3	23 ± 4^†^
Vt (mL)	454 ± 96	430 ± 123^†^	438 ± 115	367 ± 132^†^
pH	7.46 ± 0.05	7.44 ± 0.06^†^	7.47 ± 0.04	7.44 ± 0.04^†^
pCO_2_ (mmHg)	37 ± 6	38 ± 6^†^	41 ± 9	42 ± 14 †
Hb (g/dL)	9.9 ± 1.8	9.9 ± 1.8	10.1 ± 2	10.3 ± 2.1
SaO_2_ (%)	96 ± 8	97 ± 3	97 ± 3	96 ± 3
ScvO_2_ (%)	66 ± 9	66 ± 8	62 ± 9	61 ± 5*
StO_2_ (%)	80 ± 6	80 ± 7	76 ± 11	77 ± 9
DeO_2_ (%/min)	− 13.1 ± 8.4	− 13.6 ± 10.7	− 12.7 ± 7	− 17.2 ± 10.3^†^
nirVO_2_ (U)	139 ± 80	159 ± 135^†^	129 ± 75	174 ± 97^†^
THI (U)	10.7 ± 2.9	11.3 ± 3.1^†^	10.0 ± 3.9	10.5 ± 4.0
ReO_2_ (%/s)	4.1 ± 1.6	4.2 ± 1.8^†^	3.3 ± 1.9*	3.7 ± 2.0
Hyperemia AUC (U)	12.2 ± 6.5	12.9 ± 5.7	7.1 ± 4.7**	8.4 ± 4.1**
DeO_2_ ratio		1.1 ± 0.3		1.5 ± 0.6**
nirVO_2_ ratio		1.1 ± 0.4		1.5 ± 0.5*

*MV* Mechanical ventilation, *COPD* Chronic obstructive pulmonary disease, *HR* heart rate, *MAP* mean arterial pressure, *RR* respiratory rate, *Vt* tidal volume, *pCO*_*2*_ arterial partial pressure of carbon dioxide, *Hb* Hemoglobin, *SaO*_*2*_ arterial oxygen saturation, *ScvO*_*2*_ central venous oxygen saturation, *StO*_*2*_ tissue oxygen saturation, *DeO*_*2*_ StO_2_ deoxygenation rate, *nirVO*_*2*_ NIRS-derived local oxygen consumption, *THI* tissue hemoglobin index, *ReO*_*2*_ StO_2_ reoxygenation rate

* *p* < 0.05; and ** *p* < 0.01, as compared to success group at the same time point; ^†^*p* < 0.05, as compared to baseline values within the group

### StO_2_ variables

Baseline ReO_2_ and *H*_AUC_ values were significantly lower in the extubation failure group (Table 1). The weaning success group showed no significant changes in StO_2_, DeO_2_, and *H*_AUC_ after 30 min of SBT, while nirVO_2_ and THI significantly increased. The extubation failure group showed a significant decrease in their DeO_2_ slope, as well as an increase in nirVO_2_ after 30 min of SBT, while no changes in StO_2_, *H*_AUC_ and THI were observed. The DeO_2_ ratio (represented as the ratio of DeO_2_ at 30 min to baseline DeO_2_) was significantly higher in the failure group (1.5 ± 0.6 vs. 1.1 ± 0.3 in the success group, *p *< 0.01). The nirVO_2_ ratio was also higher in the failure group (1.5 ± 0.5 vs. 1.1 ± 0.4 in the success group, *p* < 0.05).

### Extubation failure prediction

Univariate analysis showed that StO_2_, ReO_2_, and *H*_AUC_ at baseline, StO_2_ and *H*_AUC_ after 30 min of the SBT, DeO_2_ ratio, and nirVO_2_ ratio were associated with extubation failure. In a multivariate analysis, only baseline StO_2_, baseline *H*_AUC_ and DeO_2_ ratio persisted significantly associated with extubation failure (Table 2).

**Table 2:** Multivariate regression analysis

	B	*ß*	*p*	(95% CI)
Baseline StO_2_	− 0.075	0.93	0.03	0.87–0.99
Baseline hyperemic AUC	− 0.192	0.83	0.001	0.74–0.92
DeO_2_ ratio	2.849	17.3	< 0.001	4.1–72.8

The AUC for predicting extubation failure for baseline StO_2_, baseline *H*_AUC_, and DeO_2_ ratio are displayed in Table 3. A logistic regression-derived StO_2_ score, combining baseline StO_2_, baseline *H*_AUC_ and DeO_2_ ratio, proved an AUC of 0.84 (95% CI 0.74–0.93, *p* < 0.001) for prediction of extubation failure. An StO_2_ score cut-off value of − 1.23 showed an LR + of 7 for predicting extubation failure (Additional file 2: Figure S1).

**Table 3:** Prediction of extubation failure

	AUC	*p*	(95% CI)
StO_2_ at baseline	0.57	ns	0.43–0.72
DeO_2_ ratio	0.72	< 0.01	0.58–0.85
nirVO_2_ ratio	0.69	0.02	0.56–0.81
Hyperemia AUC_0_	0.73	0.01	0.58–0.88
Hyperemia AUC_30_	0.74	< 0.01	0.62–0.87
StO_2_ score	0.84	< 0.001	0.74–0.93
ScvO_2_ at min 30	0.72	0.04	0.58–0.85
% change in ScvO_2_	0.59	ns	

*DeO*_*2*_*ratio* quotient between StO_2_-deoxygenation rate at the end of SBT and at baseline, *nirVO*_*2*_*ratio* quotient between NIRS-derived local oxygen consumption at the end of SBT and at baseline, *H*_AUC_ area under the curve of the hyperemic response, *StO*_*2*_ tissue oxygen saturation, *ScvO*_*2*_ central venous oxygen saturation, % change in ScvO_2_ refers to the % change in ScvO_2_ at min 30 of the SBT as compared to baseline ScvO_2_

## Discussion

Our study demonstrates that alterations in StO_2_-derived parameters were predictive of extubation failure after a clinically successful SBT.

To our knowledge, this is the first study showing that regional oxygenation parameters are associated with extubation outcome. The peripheral circulation has been previously studied in the setting of a cardiovascular stress test such as the SBT. Several studies showed that changes in gastric mucosal pH within the SBT were predictive of the clinical outcome of such SBT [12–15]. Similar results were obtained when assessing the peripheral circulation by means of NIRS, non-invasively monitoring oxygenation of the skeletal muscle within the SBT [16, 22, 23]. However, they were all small studies, and never explored the ability to predict the outcome of the overall weaning process. In this prospective study, we analyzed the utility of StO_2_-derived parameters in the extubation process, after a clinically successful SBT. Our results add new evidence on the usefulness of regional non-invasive parameters as a complimentary tool in monitoring the cardiovascular performance of critically ill patients. Indeed, our data suggest that the inclusion of StO_2_ monitoring in the weaning process might help prevent failed extubations, and therefore, the potential negative consequences on the outcome of critically ill patients.

Transitioning from positive pressure ventilation to spontaneous ventilation determines an increase in the work of breathing, and thus, an increase in the oxygen demand of the respiratory muscles. Such increase in the metabolic demand causes a sympathetic activation, in an attempt to optimize cardiac output delivery to the metabolically active tissues [24], and to increase vasomotor tone, redistributing blood flow away from the periphery toward the respiratory muscles [10, 25]. The ability of the cardiovascular system to meet this increased demand might be closely related to the ability of the patient to tolerate spontaneously breathing. Therefore, when the cardiovascular performance is limited, and/or the cost of breathing excessive, extubation failure will be more likely to occur. In these situations, the increase in sympathetic tone might be more accentuated, as the cardiovascular system continues to attempt to match cardiac output to an increasing metabolic demand. Indeed, clinical criteria for determining the success of the SBT are essential symptoms of excessive sympathetic activity (i.e., tachycardia, hypertension, agitation). A subclinical manifestation of the sympathetic activation would be the degree of peripheral vasoconstriction and/or the increase in the metabolic rate, and this is the basis for regional oxygenation measurements with NIRS.

### Increased local oxygen extraction rate

Our data confirmed that relative increases in DeO_2_ during a 30-min SBT are associated with extubation failure, independent of other respiratory and hemodynamic parameters. The observed increases in DeO_2_ within the SBT might be explained by two different, but potentially concurrent, pathophysiological mechanisms: (1) local supply–demand dependency in low or inadequate blood flow states, such as blood flow diversion from the periphery; and (2) increase in local metabolic rate.

The increase in oxygen demand of the respiratory muscles during an SBT may lead to blood flow redistribution via activation of the sympathetic–adrenal system. When accentuated, this compensatory mechanism might cause a stealing effect from the periphery [10]. Accordingly, one would expect decreases in blood content of the sensed area [19, 26], along with relative increases in the DeO_2_ rate (either due to the decreased Hb content or to the sympathetically driven increase in the metabolic rate). In our population, extubation failure was associated with significant increases in DeO_2_, but no change in THI. Conversely, the success group showed no change in DeO_2_, with significant increases in THI. Such observations suggest that cardiac output increased in those patients who succeeded the weaning process. In those patients who failed, since we did not measure stroke volume, the interpretation of the lack of changes in THI is more complex. In a model of simulated hypovolemia, Bartels et al. reported that THI measured on the thenar eminence detected slight decreases in stroke volume, but changes were minimal, as compared to measurements on the forearm [26]. Therefore, we cannot exclude some degree of stealing effect as results of a limited sensitivity of the technology. Finally, a balanced effect of increased cardiac output and peripheral vasoconstriction, resulting in no changes in THI, cannot be excluded.

NirVO_2_, an estimation of local oxygen consumption [20, 21], increased in both groups during the SBT, but the magnitude of this increase was significantly higher in the extubation failure group. NirVO_2_ differences might reflect dissimilar degrees in sympathetic activation in the setting of inadequate cardiovascular response. This hypothesis is supported by studies that showed significant increases in plasma catecholamines during the SBT, especially in patients who failed an SBT [27, 28]. Overall, our data suggest that the increase in DeO_2_ in the extubation failure group might be mainly related to an increase in local oxygen consumption, although some degree of blood flow diversion cannot be ruled out.

### Local hyperemic response after transient ischemia

We also observed that baseline endothelial performance, as measured by the StO_2_ hyperemic response, was independently associated with extubation outcome. Our findings on the predictive value of the StO_2_ hyperemic response are not surprising. Activation of the sympathetic nervous system is a major component of the neurohumoral response to the increased oxygen cost of breathing. Catecholamine release mediates vasoconstriction of peripheral vascular beds, and also, via ß-adrenoceptors, promotes vasodilation of the coronary arteries, and improves contractility. The functional impairment of the endothelium has been associated not only with poor peripheral response, but also with diminished ß-adrenoceptor sensitivity, limiting the cardiac response [29]. Therefore, endothelial dysfunction might be a potential marker of the ability of the patient to respond to the hemodynamic changes produced by the discontinuation of ventilatory support. According to our observations, we might hypothesize that vascular endothelial integrity, evaluated in peripheral skeletal muscle, might be relevant when facing a cardiovascular stress test such as transitioning from MV to spontaneously breathing.

### Study limitations

Our study has relevant limitations. First, it was carried out in a single center. Albeit we expect that similar patients should behave similarly, weaning approaches may vary across centers affecting the predictive value of these StO_2_-derived parameters. Thus, this study needs to be duplicated across other centers. Second, we did not determine the cause of weaning failure in patients who were considered to fail. We merely identified that they did fail. We can expect different behaviors of StO_2_ parameters in patients who fail because of limited cardiovascular reserve from patients who fail because of upper airway obstruction and/or impaired secretions’ management. This issue must be taken into account in future studies. Finally, we studied a heterogeneous ICU population. Since the observed predictive value of StO_2_ might vary across populations, further studies including selected homogeneous critically ill patients are required.

## Conclusions

In a mixed ICU population, non-invasive StO_2_-derived parameters at baseline, and within a 30-min SBT, were predictive of extubation outcome. Therefore, StO_2_-derived parameters may be used as indicators of global VO_2_/DO_2_ balance when facing a cardiovascular stress test, such as liberation from mechanical ventilation.

## Supplementary information

**Additional file 1: Table S1.** Suspected etiology of extubation failure.

**Additional file 2: Figure S1.** StO_2_-derived score according to extubation outcome. The cut-off value of − 1.23 is also represented.

## Data Availability

The datasets used and/or analyzed during the current study are available from the corresponding author on reasonable demand.

## Abbreviations

DeO_2_: VOT-derived StO_2_ deoxygenation rate
FiO_2_: Fraction of inspired oxygen
*H*_AUC_: StO_2_ hyperemic response
HR: Heart rate
MAP: Mean arterial pressure
MV: Mechanical ventilation
NIRS: Near-infrared spectroscopy
nirVO_2_: NIRS-derived thenar muscle oxygen consumption
P_a_CO_2_: Arterial carbon dioxide tension
PEEP: Positive end-expiratory pressure
ReO_2_: VOT-derived StO_2_ reoxygenation rate
RR: Respiratory rate
SBT: Spontaneous breathing trial
S_a_O_2_: Arterial oxygen saturation
S_cv_O_2_: Central venous oxygen saturation
S_p_O_2_: Pulse-oxymetry saturation
StO_2_: Tissue oxygen saturation
THI: Tissue hemoglobin index
VO_2_: Global oxygen consumption
VOT: Vascular occlusion test
Vt: Tidal volume
