# Supplementary material for: Thenar oxygen saturation (StO2) alterations during a spontaneous breathing trial predict extubation failure
Source: Ann Intensive Care. 2020 May 11;10:54. doi: 10.1186/s13613-020-00670-y (PMC7214564; doi:10.1186/s13613-020-00670-y)
Supplement: Supplementary file 1 — Additional file 1: Table S1. Suspected etiology of extubation failure. [file 13613_2020_670_MOESM1_ESM.doc]

**Dynamic thenar oxygen saturation (StO_2_) changes to a vascular occlusion test during a spontaneous breathing trial predict extubation failure from mechanical ventilation**

**Additional file**

**Table S1.** Suspected etiology of extubation failure

| **Suspected cause of extubation failure** | **n (%)** |
| --- | --- |
| **Upper airway obstruction**  (post-extubation stridor) | 3 (13) |
| **Cardiac failure** | 8 (35) |
| **Respiratory failure**  (respiratory muscle load and capacity imbalance) | 12 (52) |

**Figure S1.- *StO_2_-derived score* according to extubation outcome.** The cut-off value of -1.23 is also represented.
