# Supplementary figures and images for: Thenar oxygen saturation (StO2) alterations during a spontaneous breathing trial predict extubation failure
Source: Ann Intensive Care. 2020 May 11;10:54. doi: 10.1186/s13613-020-00670-y (PMC7214564; doi:10.1186/s13613-020-00670-y)

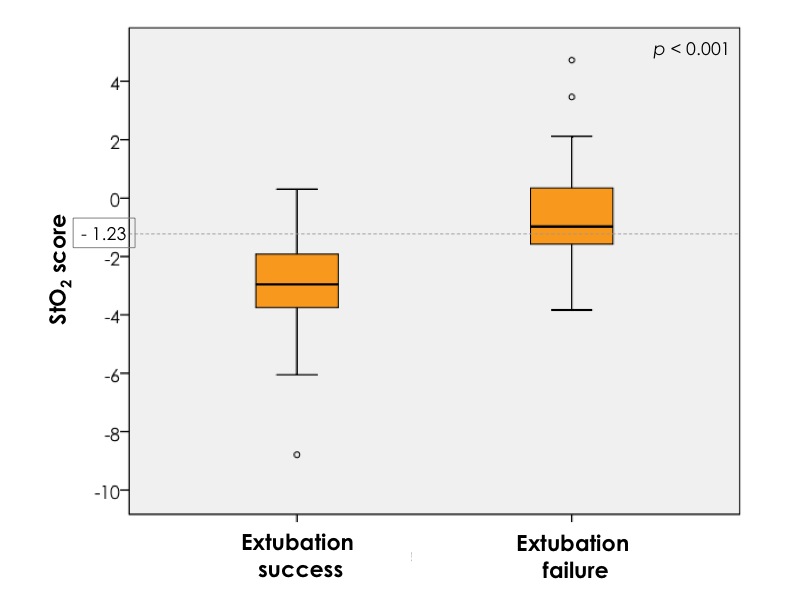

Supplement: Supplementary file 2 — Additional file 2: Figure S1. StO2-derived score according to extubation outcome. The cut-off value of − 1.23 is also represented. [file 13613_2020_670_MOESM2_ESM.jpg]
